# Site-selective ^13^C labeling of proteins using erythrose

**DOI:** 10.1007/s10858-017-0096-7

**Published:** 2017-02-28

**Authors:** Ulrich Weininger

**Affiliations:** 1 Department of Biophysical Chemistry, Center for Molecular Protein Science, Lund University, P.O. Box 124, 22100 Lund, Sweden; 2 Institute of Physics, Biophysics, Martin-Luther-University Halle-Wittenberg, 06120 Halle (Saale), Germany

**Keywords:** Relaxation, Protein dynamics, Aromatic side chain, Isotope labeling

## Abstract

NMR-spectroscopy enables unique experimental studies on protein dynamics at atomic resolution. In order to obtain a full atom view on protein dynamics, and to study specific local processes like ring-flips, proton-transfer, or tautomerization, one has to perform studies on amino-acid side chains. A key requirement for these studies is site-selective labeling with ^13^C and/or ^1^H, which is achieved in the most general way by using site-selectively ^13^C-enriched glucose (1- and 2-^13^C) as the carbon source in bacterial expression systems. Using this strategy, multiple sites in side chains, including aromatics, become site-selectively labeled and suitable for relaxation studies. Here we systematically investigate the use of site-selectively ^13^C-enriched erythrose (1-, 2-, 3- and 4-^13^C) as a suitable precursor for ^13^C labeled aromatic side chains. We quantify ^13^C incorporation in nearly all sites in all 20 amino acids and compare the results to glucose based labeling. In general the erythrose approach results in more selective labeling. While there is only a minor gain for phenylalanine and tyrosine side-chains, the ^13^C incorporation level for tryptophan is at least doubled. Additionally, the Phe ζ and Trp η2 positions become labeled. In the aliphatic side chains, labeling using erythrose yields isolated ^13^C labels for certain positions, like Ile β and His β, making these sites suitable for dynamics studies. Using erythrose instead of glucose as a source for site-selective ^13^C labeling enables unique or superior labeling for certain positions and is thereby expanding the toolbox for customized isotope labeling of amino-acid side-chains.

**Electronic supplementary material:**

The online version of this article (doi:10.1007/s10858-017-0096-7) contains supplementary material, which is available to authorized users.

## Introduction

Proteins are dynamic entities. They continuously undergo all kinds of dynamic processes on various time scales, like conformational rearrangements of the backbone, side chains and loops, ring-flips, proton transfers, changing conformations to alternative states, (partially) unfolding, domain reorientation, etc. While it is of fundamental interest to understand intrinsic protein dynamics, many of these processes are also directly linked to function (Mittermaier and Kay 2006). Fast time-scale fluctuations on the ps-ns range are connected to conformational entropy (Akke et al. 1993) and contribute to the free energy of binding or folding (Diehl et al. 2010; Frederick et al. 2007). Slower processes on the µs-ms time-scale are crucial for ligand binding (Malmendal et al. 1999), enzymatic activity (Boehr et al. 2006; Cole and Loria 2002; Eisenmesser et al. 2002) and signal transduction (Volkman et al. 2001).

NMR spectroscopy is a powerful technique to study such dynamic processes on various time-scales at atomic resolution (Palmer 2004). While the majority of studies have focused on the protein backbone using inexpensive and robust ^15^N labeling (Akke and Palmer 1996; Ishima and Torchia 2003; Jarymowycz and Stone 2006; Loria et al. 1999), more and more methods have been developed to study amino-acid side chains (Hansen and Kay 2011; Hansen et al. 2012; Lundstrom et al. 2009; Millet et al. 2002; Muhandiram et al. 1995; Mulder et al. 2002; Paquin et al. 2008). These approaches complement existing backbone studies and widen the view on certain processes, but also enable unique additional information of structure (Korzhnev et al. 2010; Neudecker et al. 2012), ring-flips (Weininger et al. 2014b), and proton occupancy and transfer reactions (Hansen and Kay 2014; Wallerstein et al. 2015). A key requirement therefore is to site-selectively label the protein, in order to generate isolated ^1^H-^13^C spin pairs (for fast dynamics also isolated ^2^H) that are not affected by coupling with their neighbours.

Aromatic residues are bulky and form a substantial part of protein hydrophobic cores. They are also over-represented in binding sites (Lo Conte et al. 1999). Especially Tyr and Trp contribute significantly to the binding free energy (Bogan and Thorn 1998). His and Tyr play important catalytic residues for enzyme activity (Bartlett et al. 2002). His can exist in three different states, one protonated and two different tautomeric neutral forms. Transient changes between these states affect hydrogen bonding patterns around the histidine. Studying ring flips of the symmetric Tyr and Phe can give insights into their packing and local transient protein breathing motions (Li et al. 1999; Wagner 1980; Wagner et al. 1976). Thus it is of great interest to monitor the dynamics of aromatic residues on both the ps-ns and µs-ms time scales.

Studies of dynamics of aromatic residues have a long history. The finding and quantification of fast ring-flips and their linkage to protein breathing motions have fundamentally changed our view on proteins (Li et al. 1999; Wagner 1980; Wagner et al. 1976). Early studies were based on proton line-shapes, which often limited the application. With easy and robust labeling protocols to achieve site-selective ^13^C labeling (Lundstrom et al. 2007; Teilum et al. 2006) studies of dynamics on aromatic side chains are undergoing a renaissance. Improved methods of obtaining relaxation rates have been developed (Weininger et al. 2012a) and the first studies of order parameters have been reported (Boyer and Lee 2008; Kasinath et al. 2015, 2013). Additionally, residual dipolar couplings have been obtained (Sathyamoorthy et al. 2013). Experiments designed to characterize dynamics on the ms (Weininger et al. 2012b) and µs (Weininger et al. 2014a) time-scales have been developed. We have recently reinvestigated the ring-flips in BPTI (Weininger et al. 2014b) using these methods, which enabled us to resolve inconsistencies between experiments (Wagner et al. 1987, 1976) and molecular dynamics simulations (Shaw et al. 2010).

While site-selective ^13^C enriched glucose (1- and 2-^13^C) has made it possible to routinely perform advanced heteronuclear studies of dynamics in aromatic side chains, its ^13^C incorporation yields are far from optimal, typically reaching 20–50%. Furthermore, it is controversial whether additional deuteration is needed (Kasinath et al. 2013) or not (Weininger et al. 2012a) in order to obtain artifact free order parameters. For µs (Weininger et al. 2014a) and ms (Weininger et al. 2012b) dynamics studies however deuteration is usually not required, but serves to prevent rather uncommon strong-coupling effects, which complicate recorded relaxation dispersions, but also contain additional information. Based on the dependence of strong-coupling effects on the refocusing frequency, slow ring-flips for degenerate chemical shifts could be identified (Weininger et al. 2013). Further, deuteration is not needed in order to get improved spectra for structural studies (Milbradt et al. 2015) or studies involving residual dipolar couplings (RDCs) (Sathyamoorthy et al. 2013).

Site-selective ^13^C enrichment using precursors other than glucose (Lundstrom et al. 2007; Teilum et al. 2006) have been developed recently. Pyruvate (Milbradt et al. 2015), 4-^13^C erythrose in combination with deuterated pyruvate (Kasinath et al. 2013), and more advanced chemically synthesized precursors for labeling of Trp (Schörghuber et al. 2015), Tyr and Phe (Lichtenecker et al. 2013), including perdeuteration of all other hydrogen positions in the aromatic side-chain. All these methods are common *in-vivo* labeling strategies using *E. coli* for protein expression. Additionally, advanced *in-vitro* strategies using the SAIL approach have been developed for Trp (Miyanoiri et al. 2011), Tyr and Phe (Takeda et al. 2010). Again, all non-^13^C labeled positions are perdeuterated.

Here we present an easy and robust approach using selectively labeled erythrose (1-, 2-, 3- and 4-^13^C) in combination with unlabeled glucose. This approach is very close to standard ^13^C labeling using glucose. The only modification is the additional presence of erythrose. Further, we quantify the ^13^C incorporation in aromatic side-chains and all other positions of the 20 amino acids for the first time and compare it to that achieved with glucose-based labeling. Erythrose labeling leads to a slight enhancement of ^13^C levels for Phe and Tyr δ, and roughly to a doubling for all proton-bound carbons in the six-ring moiety of Trp. Further the method efficiently labels Phe (and Tyr) ζ and Trp η2 (2-^13^C erythrose) and thus makes these positions available for studies of dynamics for the first time. Especially Phe ζ is of great potential interest in order to separate the effects of motions around chi-2 and chi-1 dihedral angles. Additionally, His β becomes significantly ^13^C-labeled, and Ile β, Lys β and β and Arg β become isolated ^13^C labeled. Finally, we show that the erythrose-based approach for site-selective ^13^C labeling can be easily combined with the glucose approach, allowing for more custom labeling.

## Materials and methods

### Selective ^13^C enriched isotopes

All isotopes were purchased from cortecnet. Typical prices per gram are: 1-^13^C glucose, 175 €; 2-^13^C glucose, 200 €; 1-^13^C erythrose, 450 €; 2-^13^C erythrose, 1250 €, 3-^13^C erythrose, 3400 €; 4-^13^C erythrose, 1100 €. 2 g/l glucose and 1 g/l (in case of Phe and Tyr) or 2 g/l (in case of Trp) erythrose are usually used. Up to now erythrose is only competitive in costs for desired Phe and Tyr ε* labeling (1 g/l 1-^13^C erythrose to 2 g/l 2-^13^C glucose) with similar ^13^C incorporation levels. Labeling of all other positions is more expensive with erythrose but can be justified by significantly higher ^13^C incorporation (Trp ε3, ζ3, and ζ2) or effectively labeling positions not labeled by 1-^13^C or 2-^13^C glucose (Trp η2, Phe and Tyr ζ).

### Expression and purification

An optimised coding sequence for human FK506 binding protein 12 (FKBP12; Uniprot: P62942) was synthesised (GenScript, Piscataway, NJ, USA) and sub-cloned into the plasmid pNIC28-Bsa4 (Savitsky et al. 2010).

Recombinant FKBP12 containing an N-terminal 6x His-tag tag was expressed in M9 minimal medium with 1 g/l ^15^N NH_4_Cl and 2 g/l glucose (1-^13^C, or 2-^13^C labeled, or unlabeled). In the case of erythrose labeling, site-selective ^13^C enriched erythrose (1-, 2-, 3- or 4-^13^C) was additionally present at the beginning at a concentration of 2 g/l, unless otherwise indicated. Protein expression was induced by addition of 1 mM IPTG at an OD_600_ of ~0.8. Protein expression was carried out for 18 h at 25 °C. The protein was purified on a His-trap column. Afterwards the His-tag was cleaved by Tobacco Etch Virus (TEV) protease. The protein was dialysed, and collected as the flow through of another His-trap column. At the end the buffer was exchanged to NMR buffer and the protein was concentrated to ~12 mg/ml.

### NMR spectroscopy

All spectra were run on 800 µM samples containing 25 mM sodium phosphate, pH 7.0 and 10% (v/v) D_2_O at 25 °C and a static magnetic field strength of 14.1 T. For each sample, a ^1^H–^15^N plane of an HNCO, non-constant time ^1^H–^13^C HSQCs for the aliphatic and aromatic regions, and a 1D spectrum on ^13^C were recorded for quantification of ^13^C incorporation. Intensities of different samples (with possible slightly different concentration) were referenced to the averaged intensities of a ^1^H–^15^N HSQC. Assignments were checked using standard 3D experiments. Aromatic ^13^C relaxation studies were performed using L-optimized TROSY detected relaxation experiments (Weininger et al. 2012a). All spectra were processed using NMRPipe (Delaglio et al. 1995) and analysed using NMRView (Johnson 2004).

### Data analysis

The analysis was restricted to well resolved signals that only arise from the same kind of atom (residue type and position). For the fully ^13^C-enriched reference sample, volumes from both peaks split by the ^13^C–^13^C ^1^J coupling were added. All positions of interest described in this article resulting from erythrose labeling (and glucose labeling for comparison) were isolated and showed no signs of any ^13^C–^13^C ^1^J coupling. Intensities were normalized to the fully ^13^C enriched sample and expressed in %. By analysing multiple signals of the same kind, the relative error in the intensities of ^13^C covalently bound to ^1^H could be estimated to 1%. Errors for ^13^C not bound to ^1^H were estimated to 3%.

## Results and discussion

**Fig. 1:**
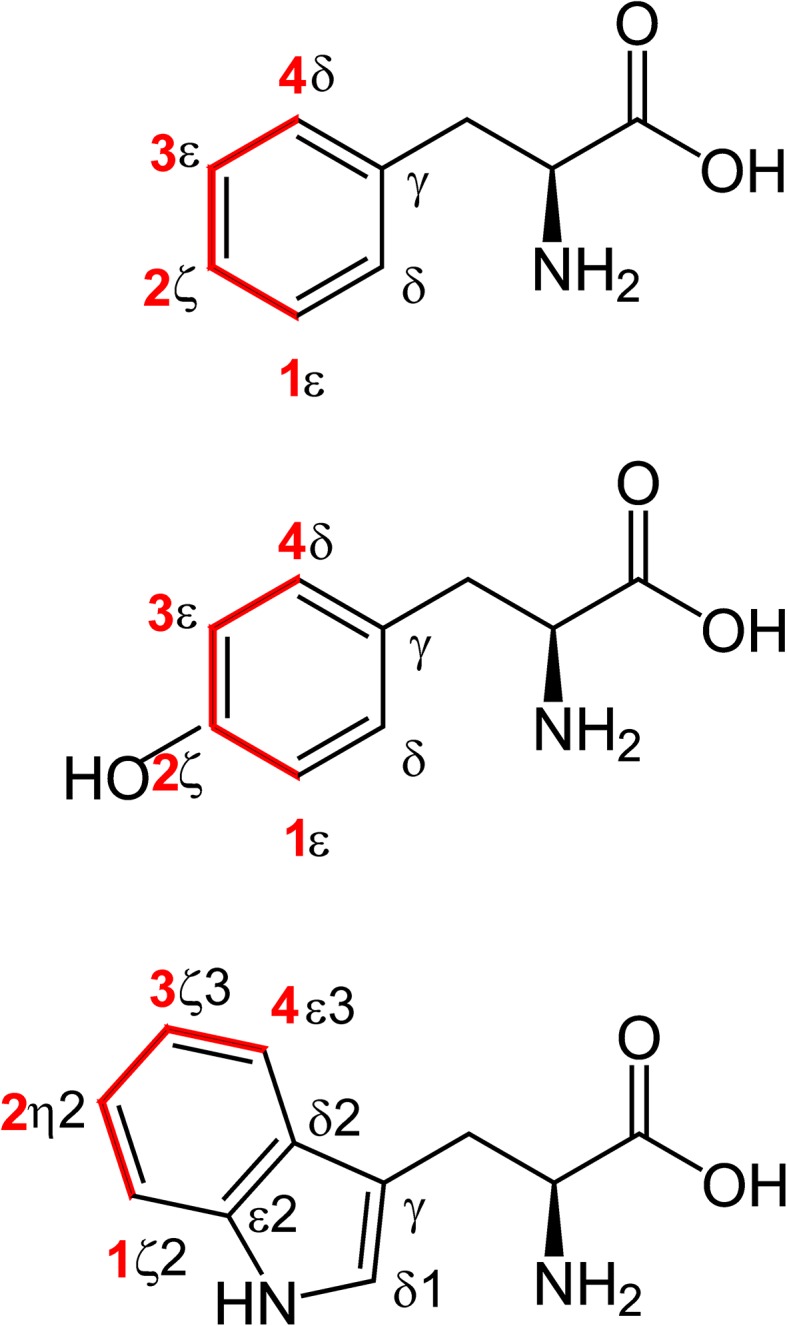
Site-selective ^13^C incorporation in aromatic side-chains using site-selectively labeled erythrose. Phenylalanine, tyrosine and tryptophan (from *top* to *bottom*) are shown with the positions in the aromatic rings labeled. Incorporation of carbons from erythrose is shown in *red*, with the positions of erythrose (1–4) labeled. Other positions (shown in *black*) can be labeled if erythrose is scrambling into other pathways

Erythrose is a precursor that enters the metabolic pathways closer to the amino-acid product than does glucose, which is of great advantage for achieving site-selective ^13^C labeling of aromatic side chains in proteins (Fig. 1). To make the labeling procedure as generally applicable and simple as possible, ^13^C-labeled erythrose (1-, 2-, 3- or 4-^13^C) was added together with unlabeled glucose to the minimal medium, ensuring that the growth rate of *E. coli* is essentially the same as for standard minimal media conditions. Furthermore, this approach allows for combined ^13^C labeling by erythrose and glucose. Preliminary tests showed that adding the erythrose at the very beginning does not lead to any scrambling in the aromatic side chains compared to the result obtained when adding it shortly before induction. Since the level of ^13^C incorporation is slightly higher when erythrose is added at the start this procedure was followed in all experiments. The level of ^13^C incorporation was monitored for all aromatic side-chains, with exception of Tyr γ, His γ, and Trp δ2 and ε2, as well as for all other carbon sites in the 20 amino acids. All the missing positions do not have any attached proton. The resulting data provides information on background labeling, scrambling, and unexpected selective incorporations, as described below.

### Site-selective ^13^C labeling of aromatic side-chains

**Fig. 2:**
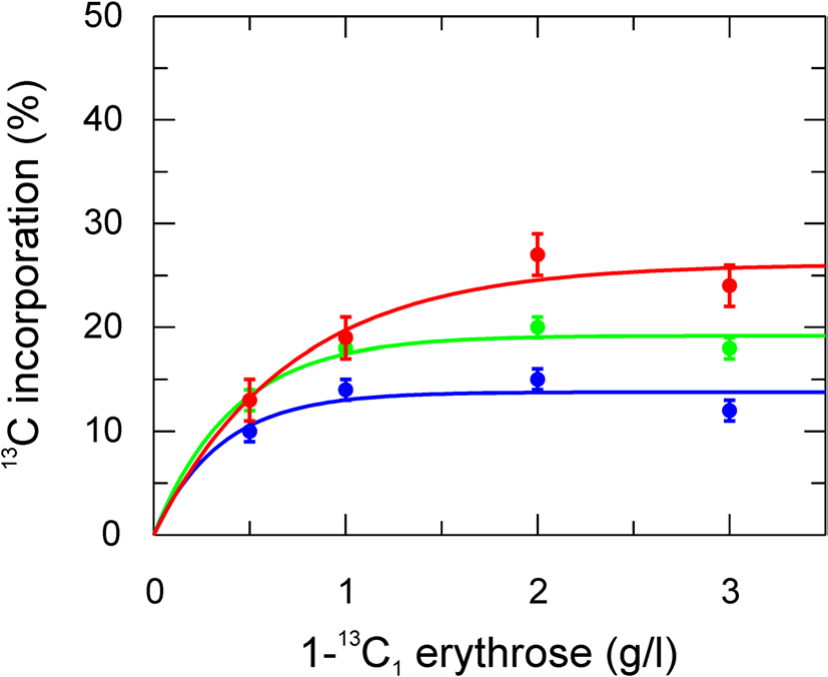
^13^C incorporation level in aromatic side-chains resulting from different amounts of 1^13^C erythrose in the medium. Incorporation in Phe ε* (*green*), Tyr ε* (*blue*) and Trp ζ2 red are shown in % relative to fully ^13^C enriched glucose. *Solid lines* are single exponential fits. Note that for Phe and Tyr the referencing is for two carbons (1 and 2, represented by* asterisk*), since the aromatic rings undergo fast ring-flips on the NMR time-scale

The above mentioned erythrose labeling strategy leads to following general observations. In aromatic side-chains isolated ^13^C labeling occurs at the expected positions (Fig. 1) and the background labeling of other positions is less than that obtained using glucose as the sole carbon source. Next, the optimal amount of labeled erythrose in the expression medium was tested using different amounts of 1-^13^C_1_-erythrose (Fig. 2). Phe and Tyr reach a maximum in ^13^C incorporation already at 1 g erythrose per liter medium, whereas for Trp the level increases to 2 g/l. Since signals from Trp are weaker in general (in Phe and Tyr two positions normally contribute to the same signal for δ and ε because of fast ring-flips), 2 g/l erythrose were used for the following study. However, if one is only interested in Phe and Tyr, 1 g/l should be enough.

**Fig. 3:**
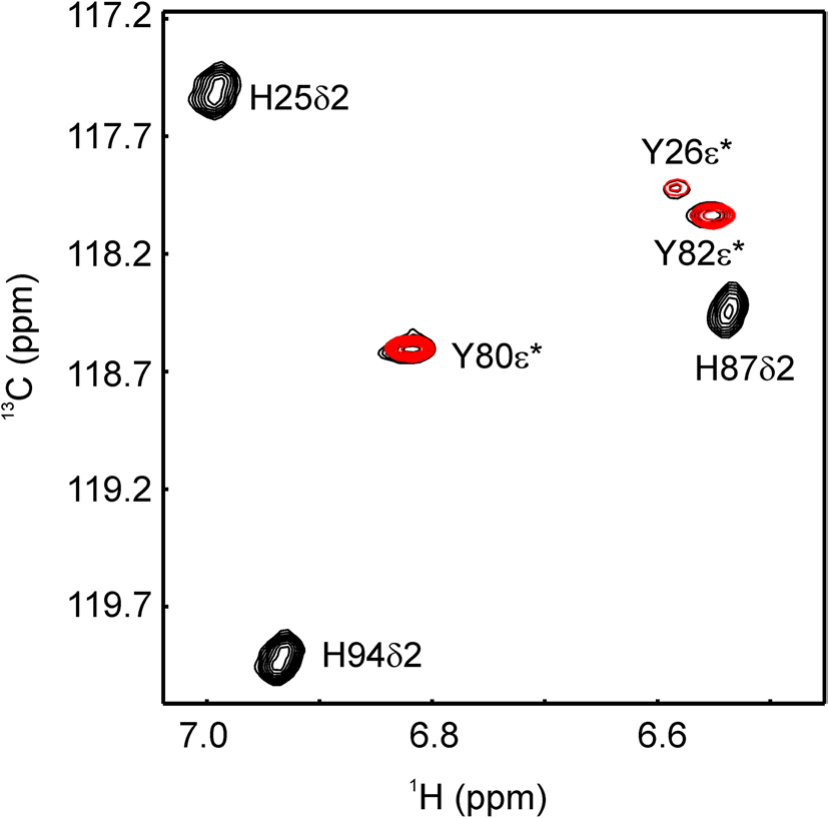
Tyr ε* His δ2 region of an aromatic ^1^H^13^C-TROSY-HSQC of FKBP12. Signals arising from a 2-^13^C_1_-glucose labeled sample are shown in *black*, while signals from a 1-^13^C_1_-erythrose labeled sample are shown in red. His δ2 signals are broadened because ^15^N was not decoupled.* Asterisk* represents an averaged signal of position 1 and 2 because of fast exchange of the aromatic rings on the NMR time-scale

^13^C incorporation levels in Phe, Tyr and Trp using differently labeled erythrose or glucose are summarized in Table 1 (incorporation levels for all positions and amino acids using erythrose labeling are listed in SI Tables 1, 2). All ^13^C labeled positions do not show any signs of ^13^C–^13^C couplings in the spectra (SI Fig. 1) in agreement with the low ^13^C incorporation for neighbored positions (Table 1). For Phe and Tyr, erythrose (4-^13^C) labeling leads to a higher incorporation yield in position δ. Additionally position ζ becomes accessible (with 2-^13^C erythrose), which is potentially very useful to differentiate fluctuations around chi-2 from fluctuations around chi-1, or ring flips from general conformational exchange. For the ε position the ^13^C incorporation level is very similar for the two carbon sources (1-^13^C or 3-^13^C erythrose, or 2-^13^C glucose). As for Trp, position δ1 is not labeled at all, which is expected. In case of Trp ε3, ζ3 and ζ2, erythrose (4-^13^C, 3-^13^C, and 1-^13^C) yields at least twice as high ^13^C incorporation. Additionally η2 becomes efficiently labeled by 2-^13^C erythrose. Since His δ2 is not labeled (analogously to Trp δ1), erythrose (1-^13^C and 3-^13^C) labeling allows for studies on Tyr ε without potential disturbance of His δ2, which shares the same spectral region (Fig. 3). This could be of particular interest for studies of ring flips where Tyr ε signals might be broad and are harder to track.

**Table 1:** Site-selective ^13^C incorporation in aromatic side-chains using glucose (G) or erythrose (E)

	1-^13^C G	2-^13^C G	1-^13^C E	2-^13^C E	3-^13^C E	4-^13^C E
Phe γ	6	55	2	4	24	0
Phe δ*	34	4	1	2	1	41
Phe ε*	2	22	20	4	23	1
Phe ζ	1	1	5	39	1	1
Tyr γ	n.d	n.d	n.d	n.d	n.d	n.d
Tyr δ*	32	4	2	2	1	45
Tyr ε*	1	19	17	3	23	1
Tyr ζ	0	0	7	48	11	5
Trp γ	9	10	1	3	0	1
Trp δ1	26	49	4	3	2	2
Trp δ2	n.d	n.d	n.d	n.d	n.d	n.d
Trp ε2	n.d	n.d	n.d	n.d	n.d	n.d
Trp ε3	26	2	1	2	1	54
Trp ζ3	1	24	1	1	52	1
Trp η2	1	2	6	35	1	0
Trp ζ2	2	12	27	5	0	1

Values are in %. Errors are estimated to 1% for ^1^H bound ^13^C, 3% for others. 1% for non labeled positions is expected because of natural abundance of ^13^C

*Represents an averaged signal of position 1 and 2 because of fast exchange of the aromatic rings on the NMR time-scale

**Table 2:** Site-selective ^13^C incorporation in histidine using glucose (G) or erythrose (E)

	1-^13^C G	2-^13^C G	1-^13^C E	2-^13^C E	3-^13^C E	4-^13^C E
His CO	31	7	1	0	0	3
His α	3	40	1	1	24	1
His β	0	0	9	34	1	0
His γ	n.d	n.d	n.d	n.d	n.d	n.d
His δ2	26	52	4	4	3	2
His ε1	37	8	1	1	3	14

Values are in %. Errors are estimated to 1% for ^1^H bound ^13^C, 3% for others. 1% for non labeled positions is expected because of natural abundance of ^13^C

While the experimental ^13^C incorporation (Table 1) of 1-^13^C and 2-^13^C erythrose is closely following the expected incorporation (Fig. 1, red), 3-^13^C and 4-^13^C erythrose show some signs of scrambling and labeling of unexpected positions (Fig. 1, black). For 3-^13^C erythrose ^13^C is ending in Phe γ, and for 4-^13^C erythrose the incorporation of ^13^C in the δ positions (40% of δ1 and δ2) is higher than for the ε (20% of ε1 and ε2) and z (40% of only one ζ). It is unclear if the additional non expected ^13^C (Fig. 1, black) end up in the same molecule as the expected (Fig. 1, red), causing possible ^2^J ^13^C-^13^C couplings, or in different molecules. Please note, that this is the default situation in glucose labeling. On the other hand, the absence of effective ^13^C incorporations in other positions than the expected, in case of 1-^13^C and 2-^13^C erythrose clearly indicates the absence of any possible ^2^J or ^3^J ^13^C-^13^C couplings.

### ^13^C relaxation studies

**Fig. 4:**
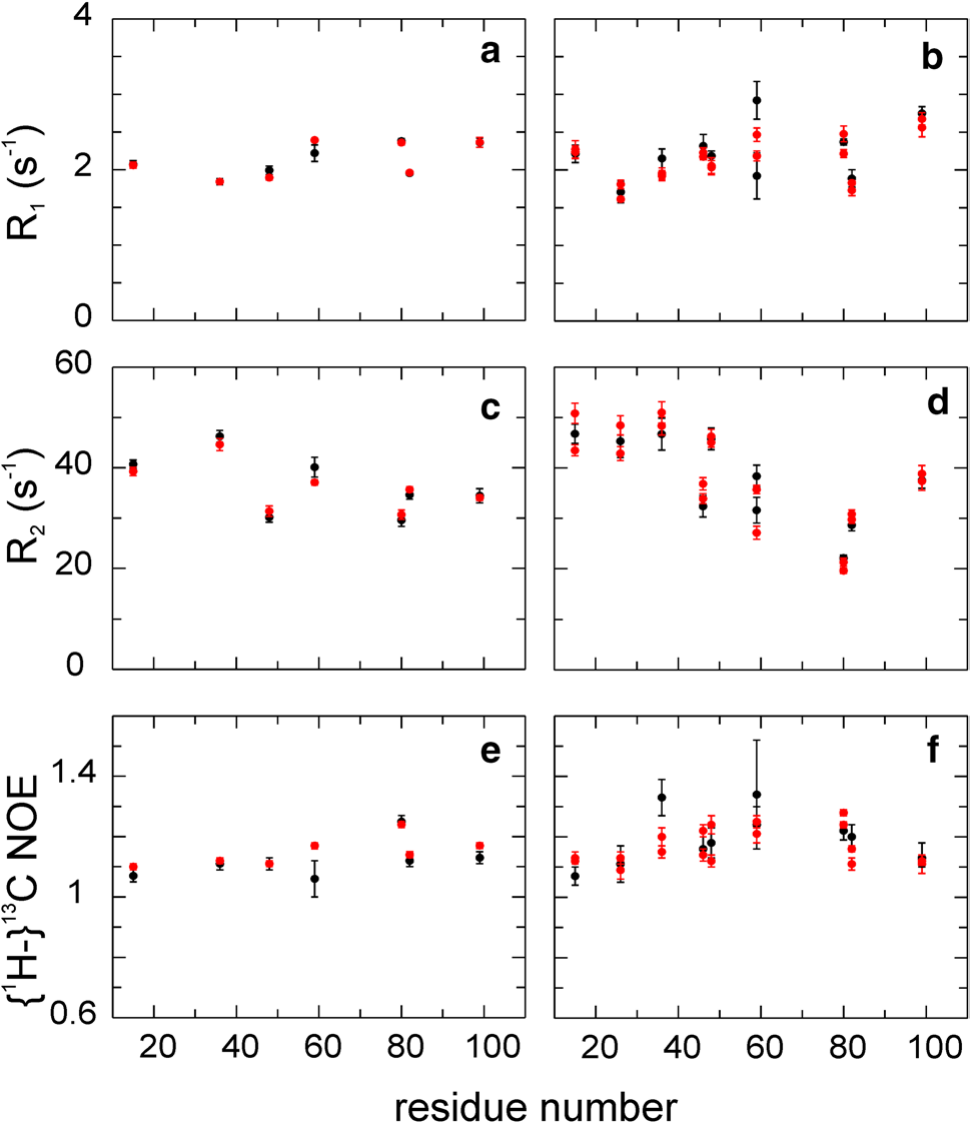
Comparison of aromatic ^13^C relaxation experiment using glucose (1-^13^C and 2-^13^C) or erythrose (1-^13^C, 3-^13^C, and 4-^13^C) labeled FKBP12. *R*_1_ (**a, b**), *R*_2_ (**c, d**) and {^1^H-}^13^C NOE (**e**,** f**) experiments were conducted using site-selective labeled FKBP12 based on glucose (*black*) or erythrose (*red*) labeling. Results from 4 to ^13^C_1_-erythrose and corresponding sites from glucose are shown in (**a, c, e**), results from 1 to ^13^C_1_- and 3-^13^C_1_-erythrose and corresponding sites from glucose are shown in (**b, d, f**)

Both erythrose and glucose labeling lead to site-selective ^13^C labeling in aromatic side-chains. Are they both equally well suited for ^13^C relaxation studies or are potential long-range ^13^C-^13^C couplings affecting the results? Since erythrose labeling leads to less ^13^C background in the protein and a more distinct labeling of the aromatic side-chains, potential problems are expected to be less. However, comparing *R*_1_ (SI Fig. 2), *R*_2_ (SI Fig.3) and {^1^H-}^13^C NOE for identical positions between erythrose- (1-^13^C, 3-^13^C, and 4-^13^C) and glucose- (1-^13^C and 2-^13^C) labeled samples, we observe an excellent agreement (Fig. 4). Thus, the two approaches give virtually the same result; small deviations do not follow any trend indicating systematic differences, but appear to be random. Only for poorly ^13^C labeled positions obtained with glucose labeling (Trp), the relaxation data are slightly different, which can be explained by the higher uncertainty of the glucose-based probe (due to the lower S/N).

While this does not clarify if additional deuteration is needed for artifact free relaxation data (Kasinath et al. 2013) or not (Weininger et al. 2012a), it shows that remote ^13^C do not play a role and potentially any method resulting in isolated ^13^C is equally well suited for relaxation studies. ^13^C relaxation dispersion experiments both for CPMG (Weininger et al. 2012b) and *R*_1ρ_ (Weininger et al. 2014a) were validated for glucose labeled samples previously. These experiments can be directly applied to samples resulting from erythrose labeling, since the relaxation behaviour is identical.

### Site-selective ^13^C labeling of aliphatic side-chains

Labeling with erythrose is more selective then glucose-based labeling, since it is a precursor closer to the aromatic side-chain end products. Therefore it is not surprising that the level of ^13^C incorporation in aliphatic side-chains is generally lower (SI Tables 1, 2). However, a few positions are worth mentioning, which become efficiently labeled with isolated ^13^C. First, in histidine the α and β positions are significantly labeled (Table 2) by 3-^13^C and 2-^13^C erythrose, indicating a crossover into the pentose-5-phosphate pathway. Indeed erythrose 4-phosphate can be transformed to ribose 5-phosphate via sedoheptulose 7-phosphate by transaldolase and transketolase. (Schwender et al. 2003) In contrast, there is no ^13^C incorporation in the aromatic moiety of His. Especially His β is of potential interest, where the ^13^C incorporation is fairly significant at 34%. Since this site is not ^13^C labeled at all using 1-^13^C and 2-^13^C glucose, information in relaxation dispersion studies on the β carbon was missing (Lundstrom et al. 2009). The situation is similar for Ile and Lys β, which both are ^13^C labeled at 21% (SI Table 2 by 3-^13^C and 4-^13^C erythrose. They are efficiently labeled in the glucose (2-^13^C and 1-^13^C) approach as well, but not free from ^13^C–^13^C couplings. Furthermore, Lys δ and Arg γ are labeled at 22 and 16%, respectively, in an isolated fashion (SI Table 2), by 4-^13^C erythrose. These might be of interest as additional positions for dynamics studies in long and charged side-chains.

### Combined ^13^C labeling using both erythrose and glucose

Since the general labeling protocol presented here is based on site-selectively ^13^C-labeled erythrose in addition to unlabeled glucose, it is straightforward to combine site-selective labeling from both sources in order to get more positions per sample labeled or to increase ^13^C labeling of some sites. This strategy was verified by two approaches.

**Fig. 5:**
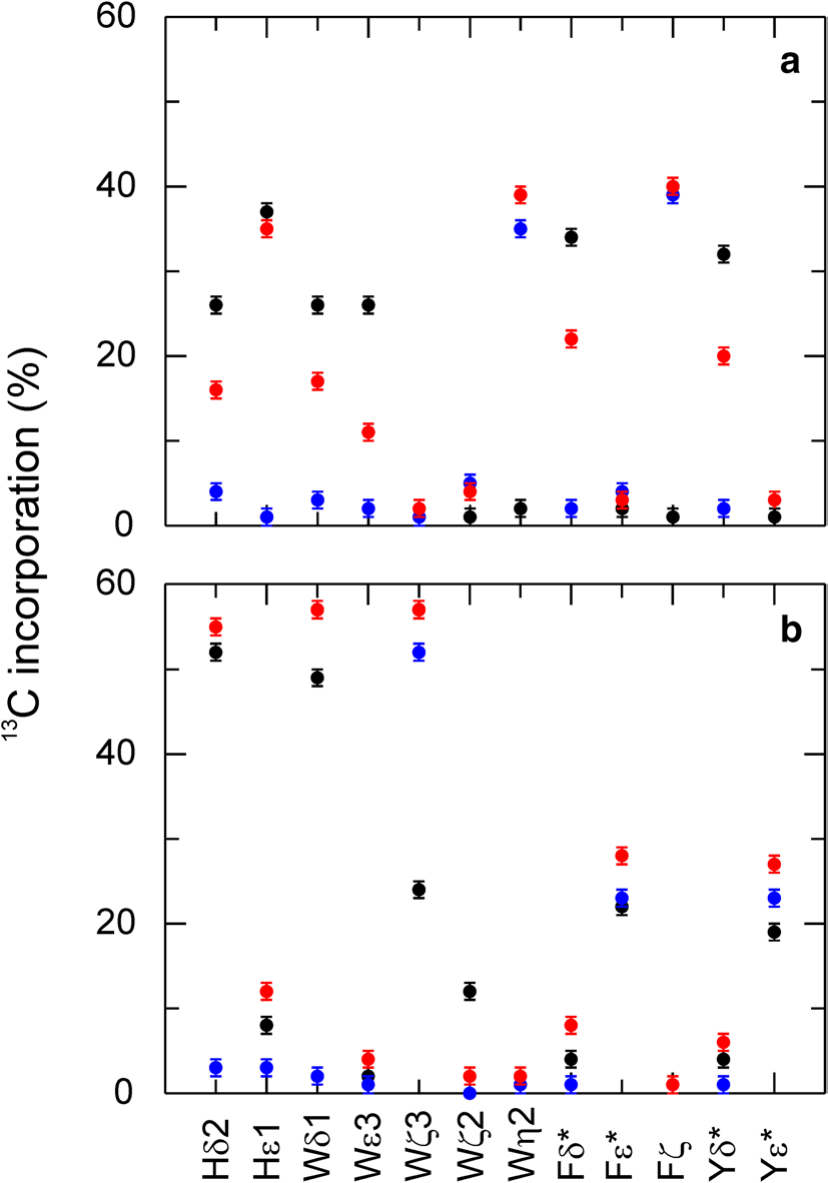
^13^C incorporation in aromatic side-chains using a combined glucose erythrose approach. Incorporation from glucose only is shown in *black*, incorporation from erythrose only in blue, labeling from both erythrose and glucose in *red*. **a** Shows results from 1 to ^13^C_1_-glucose and 2-^13^C_1_-erythrose, **b** from 2 to ^13^C_1_-glucose and 3-^13^C_1_-erythrose. All ^13^C labeled positions are isolated, no signs from ^13^C-^13^C couplings could be detected. * represents an averaged signal of position 1 and 2 because of fast exchange of the aromatic rings on the NMR time scale

First, we combined 1-^13^C_1_-glucose, which labels Phe and Tyr δ, His ε1 and δ2 and Trp δ1 and ε3 (Fig. 5a, black), with 2-^13^C_1_-erythrose, which labels Phe (and Tyr) ζ and Trp η2 (Fig. 5a, blue), positions that are not covered by glucose (1-^13^C and 2-^13^C) labeling. The combined approach (Fig. 5a, red) gives the following results. His ε1 becomes as efficiently labeled as with protocols using only glucose and Phe ζ and Trp η2 as efficiently as with erythrose. His δ2 and Trp δ1 are labeled less than in the 1-^13^C_1_-glucose-only case. However these positions are better studied with the 2-^13^C_1_-glucose approach, which results in much higher ^13^C. Phe and Tyr δ are also labeled less than with glucose labeling. However, these sites are still labeled at a reasonable level, similar to what glucose labeling achieves for Phe and Tyr ε. Since the δ signals arise from two identical positions (due to fast ring flips), they are of the same signal strength as the Phe ζ signals. The only real drawback is observed for Trp ε3, whose labeling is rather poor in the glucose approach but even worse combined with erythrose. However, the combined approach is ideal to study the δ and ζ positions of Phe and Tyr in a single sample, because the spectral regions are well separated.

Second, we combined 2-^13^C_1_-glucose, which labels Phe and Tyr ε, His δ2 and Trp δ1, ζ3 and ζ2 (Fig. 5b, black), with 3-^13^C_1_-erythrose, which also labels Phe and Tyr ε and Trp ζ3 (Fig. 5b, blue). Replacing 3-^13^C_1_-erythrose with 1^13^C_1_-erythrose gives very similar results, only replacing erythrose based labeling of Trp ζ3 by Trp ζ2. Combining 1-^13^C_1_- and 3-^13^C_1_-erythrose would label both Trp positions but only half as effective. These approaches were not tested experimentally however. The combined approach of 2-^13^C_1_-glucose and 3-^13^C_1_-erythrose (Fig. 5b, red) labels His δ2 and Trp δ1 slightly better than what is observed for glucose only. Trp ζ3 and Phe and Tyr ε have improved ^13^C levels than with either of the two single-label approaches. However, the gain is less than what is expected from theoretical considerations, which suggest levels slightly higher than 60% (Trp ζ3) or 30% (Phe and Tyr ε). As expected the ^13^C level in Trp ζ2 decreases. This approach leads to results similar to that observed when using 2-^13^C_1_-glucose only, but with a moderate increase in ^13^C levels for Phe and Tyr ε (if sensitivity is crucial) and a large increase for the Trp ζ3 (3-^13^C_1_-erythrose) or Trp ζ2 (1-^13^C_1_-erythrose).

There are various other possible combinations, as long as glucose and erythrose do not result in covalent ^13^C-^13^C neighbors (Fig. 1, 1-^13^C and 4-^13^C result in ^13^C labeling next to positions that can be labeled by glucose according to Table 1). For instance, combining 1-^13^C_1_-glucose and 4-^13^C_1_-erythrose should result in the highest ^13^C incorporation for Phe and Tyr δ and Trp ε3.

**Fig. 6:**
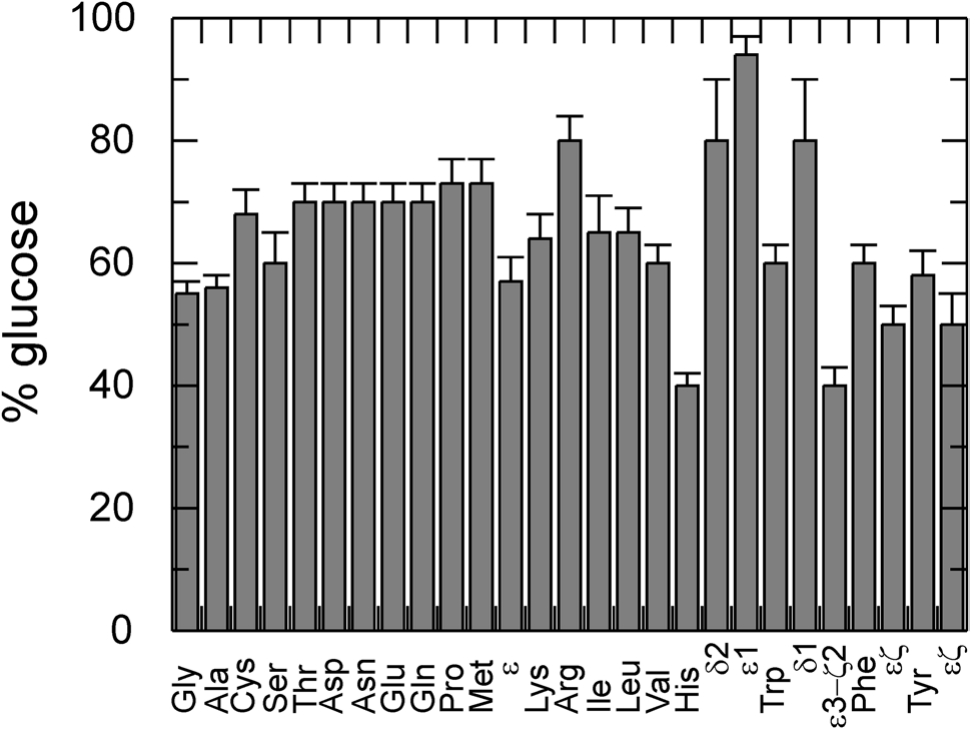
Amount of incorporation arising from glucose when erythrose is present for all amino acids. Both from individual (1-^13^C, 2-^13^C, 3-^13^C, and 4-^13^C erythrose, 1-^13^C glucose, and 2-^13^C glucose) and combined (1-^13^C glucose with 2-^13^C erythrose, 2-^13^C glucose with 3-^13^C erythrose) labeling approaches the amount of incorporation from glucose could be determined. The amount arising from erythrose is the complementary fraction. This scenario uses 2 g/l glucose and 2 g/l erythrose. Backbone carbonyl incorporation behaves differently. Whenever results from all other positions of an amino acid are in agreement with each other only one value per amino acid is shown. If certain positions of amino acids show significantly different behaviour than the rest they are shown right from the amino acids and are labeled according to their position (for Met, His, Trp, Phe and Tyr)

Based on the ^13^C incorporation levels achieved with the erythrose-only approach (1-^13^C to 4-^13^C) and the combined erythrose–glucose approaches described above, one can estimate to what extent a certain amino acid is built from glucose and erythrose precursors (Fig. 6). The following results are based on experiments using 2 g/l of each carbon source, both present in the expression medium at the beginning. For most amino acids, 60% for the carbon incorporation originates from glucose and 40% from erythrose. While this result is close to the expected 50/50 distribution according to the amount in the medium, it does not agree with the result from studies with varying erythrose concentrations (Fig. 2). The highest amount of glucose based synthesis is 80% or more, which is observed for Arg, the aromatic moiety of His and the aromatic 5-ring moiety of Trp. The lowest amount of glucose based synthesis (and thereby the highest amount of erythrose based) is observed for the aliphatic moiety of His, the aromatic 6-ring moiety of Trp with 40%, and Phe and Tyr ε and ζ with 50%.

### Further improvements

^13^C labeling of aromatic (and other) side-chains based on site-selectively ^13^C-enriched erythrose together with unlabeled glucose enables similar growth of cells as that resulting from growth on glucose only, and similar or improved ^13^C incorporation with a higher selectivity. However, labeling yields are far from 100%, which leaves room for further improvement. One way to increase the labeling yield would be to use cells with improved erythrose uptake. This will likely shift the ratio of amino acid biosynthesis more to the erythrose-based side. However, this would most likely come at the price of reduced selectivity. A more straightforward approach would be to use doubly ^13^C-enriched erythrose, which unfortunately does not appear to be commercially available at present. As long as the two ^13^C sites are separated in the erythrose they will lead to isolated ^13^C sites in the aromatic side-chains with the same level of incorporation as that obtained with the singly ^13^C-labeled erythrose. 1,3-^13^C_2_-erythrose would double the ^13^C incorporation of Phe and Tyr ε and label Trp ζ3 and ζ2 at the same time. 2,4-^13^C_2_-erythrose would label Phe and Tyr δ and ζ, and Trp ε3 and η2 at the same time. 1,4-^13^C_2_-erythrose would label Phe and Tyr δ and ε, but in this case the ^13^C sites are not expected to be isolated. Since the ^13^C incorporation in Phe and Tyr δ for 4-^13^C erythrose is higher (Table 1) than for ε and ζ (only one carbon), the other δ (Fig. 1, black) must be labeled as well by 4-^13^C_1_-erythrose, which will lead to ^13^C-^13^C couplings between δ (from 4 to ^13^C erythrose, Fig. 1, black) and ε (from 1 to ^13^C erythrose, Fig. 1, red).

## Conclusions

We have shown that erythrose as a source for site-selective ^13^C labeling of amino acids yields more selective incorporation patterns than what is achieved using glucose. Erythrose leads to a slight improvement of the ^13^C level for Phe and Tyr δ, and a significant improvement (doubling) for proton-bound carbons in the six ring moiety of Trp. Further Phe (and Tyr) ζ and Trp η2 become available for measuring dynamics for the first time. Labeling of Phe ζ make it possible to separate the effects of motions around chi-2 and chi1 dihedral angles. His β becomes significantly ^13^C labeled via erythrose, and isolated ^13^C appears in the Ile β, Lys β and δ, and Arg γ sites. Finally, we have shown that the present approach for site-selective ^13^C labeling can be easily combined with the glucose-based approach, to yield labeling patterns optimized for specific purposes.

## Electronic supplementary material

Below is the link to the electronic supplementary material.

Supplementary material 1 (DOCX 4198 KB)
