# Supplementary material for: Site-selective 13C labeling of proteins using erythrose
Source: J Biomol NMR. 2017 Feb 28;67(3):191–200. doi: 10.1007/s10858-017-0096-7 (PMC5388708; doi:10.1007/s10858-017-0096-7)
Supplement: Supplementary file 1 — Supplementary material 1 (DOCX 4198 KB) [file 10858_2017_96_MOESM1_ESM.docx]

**Site-selective ^13^C labeling of Proteins Using Erythrose**

**Ulrich Weininger**^1,2^

^1^Department of Biophysical Chemistry, Center for Molecular Protein Science, Lund University, P.O. Box 124, SE-22100 Lund, Sweden

^2^Institute of Physics, Biophysics, Martin-Luther-University Halle-Wittenberg, D-06120 Halle (Saale), Germany

e-mail: ulrich.weininger@physik.uni-halle.de

phone: +49 345 55 28555

fax: +49 345 55 27161

**SI Table 1**: Site-selective ^13^C incorporation using erythrose for Phe, Tyr, Trp, His, Gly, Ala, Ser, Cys, Thr, Met and Pro.

| position | 1-^13^C | 2-^13^C | 3-^13^C | 4-^13^C | position | 1-^13^C | 2-^13^C | 3-^13^C | 4-^13^C |
| --- | --- | --- | --- | --- | --- | --- | --- | --- | --- |
|  |  |  |  |  |  |  |  |  |  |
| Phe CO | 13 | 2 | 0 | 0 | Gly CO | 11 | 2 | 0 | 0 |
| Phe α | 2 | 1 | 26 | 3 | Gly α | 1 | 2 | 19 | 3 |
| Phe β | 1 | 1 | 1 | 26 |  |  |  |  |  |
| Phe γ | 2 | 4 | 24 | 0 | Ala CO | 11 | 3 | 0 | 0 |
| Phe δ* | 1 | 2 | 1 | 41 | Ala α | 1 | 2 | 27 | 2 |
| Phe ε* | 20 | 4 | 23 | 1 | Ala β | 2 | 2 | 3 | 37 |
| Phe ζ | 5 | 39 | 1 | 1 |  |  |  |  |  |
|  |  |  |  |  | Ser CO | 12 | 2 | 0 | 0 |
| Tyr CO | 12 | 2 | 0 | 0 | Ser α | 3 | 2 | 25 | 5 |
| Tyr α | 1 | 3 | 33 | 0 | Ser β | 1 | 2 | 4 | 30 |
| Tyr β | 1 | 2 | 1 | 34 |  |  |  |  |  |
| Tyr γ | n.d. | n.d. | n.d. | n.d. | Cys CO | 11 | 2 | 0 | 0 |
| Tyr δ* | 2 | 2 | 1 | 45 | Cys α | 2 | 2 | 15 | 2 |
| Tyr ε* | 17 | 3 | 23 | 1 | Cys β | 1 | 2 | 2 | 20 |
| Tyr ζ | 7 | 48 | 11 | 5 |  |  |  |  |  |
|  |  |  |  |  | Thr CO | 6 | 0 | 1 | 1 |
| Trp CO | 10 | 2 | 0 | 0 | Thr α | 1 | 11 | 8 | 14 |
| Trp α | 1 | 2 | 22 | 2 | Thr β | 1 | 2 | 11 | 14 |
| Trp β | 2 | 2 | 4 | 34 | Thr γ2 | 4 | 4 | 10 | 18 |
| Trp γ | 1 | 3 | 0 | 1 |  |  |  |  |  |
| Trp δ1 | 4 | 3 | 2 | 2 | Met CO | 4 | 0 | 0 | 0 |
| Trp δ2 | n.d. | n.d. | n.d. | n.d. | Met α | 1 | 2 | 6 | 6 |
| Trp ε2 | n.d. | n.d. | n.d. | n.d. | Met β | 1 | 2 | 5 | 13 |
| Trp ε3 | 1 | 2 | 1 | 54 | Met γ | 3 | 2 | 8 | 9 |
| Trp ζ3 | 1 | 1 | 52 | 1 | Met ε | 1 | 2 | 4 | 24 |
| Trp η2 | 6 | 35 | 1 | 0 |  |  |  |  |  |
| Trp ζ2 | 27 | 5 | 0 | 1 | Pro CO | 5 | 0 | 1 | 1 |
|  |  |  |  |  | Pro α | 1 | 2 | 5 | 16 |
| His CO | 1 | 0 | 0 | 3 | Pro β | 1 | 1 | 7 | 10 |
| His α | 1 | 1 | 24 | 1 | Pro γ | 2 | 2 | 2 | 16 |
| His β | 9 | 34 | 1 | 0 | Pro ε | 1 | 2 | 18 | 2 |
| His γ | n.d. | n.d. | n.d. | n.d. |  |  |  |  |  |
| His δ2 | 4 | 4 | 3 | 2 |  |  |  |  |  |
| His ε1 | 1 | 1 | 3 | 14 |  |  |  |  |  |
|  |  |  |  |  |  |  |  |  |  |

Values are in %. Errors are estimated to 1 % for ^1^H bound ^13^C, 3 % for others. 1 % for non labeled positions is expected because of natural abundance of ^13^C.

**SI Table 2**: Site-selective ^13^C incorporation using erythrose for Asn, Asp, Gln, Glu, Lys, Arg, Val, Leu and Ile.

| position | 1-^13^C | 2-^13^C | 3-^13^C | 4-^13^C | position | 1-^13^C | 2-^13^C | 3-^13^C | 4-^13^C |
| --- | --- | --- | --- | --- | --- | --- | --- | --- | --- |
|  |  |  |  |  |  |  |  |  |  |
| Asn CO | 5 | 0 | 1 | 1 | Val CO | 11 | 2 | 0 | 0 |
| Asn α | 1 | 1 | 4 | 9 | Val α | 1 | 2 | 19 | 3 |
| Asn β | 2 | 2 | 6 | 17 | Val β | 2 | 2 | 20 | 5 |
| Asn γ | -2 | -5 | 25 | 12 | Val γ* | 1 | 2 | 2 | 27 |
|  |  |  |  |  |  |  |  |  |  |
| Asp CO | 4 | 1 | 1 | 1 | Leu CO | 1 | 0 | 1 | 0 |
| Asp α | 0 | 2 | 6 | 13 | Leu α | 1 | 1 | 1 | 12 |
| Asp β | 1 | 1 | 4 | 16 | Leu β | 1 | 2 | 18 | 2 |
| Asp γ | -2 | -5 | 25 | 12 | Leu γ | 2 | 2 | 18 | 3 |
|  |  |  |  |  | Leu δ* | 2 | 2 | 2 | 28 |
| Gln CO | 4 | 0 | 1 | 1 |  |  |  |  |  |
| Gln α | 2 | 1 | 3 | 14 | Ile CO | 5 | 1 | 1 | 1 |
| Gln β | 2 | 2 | 9 | 13 | Ile α | 1 | 4 | 7 | 10 |
| Gln γ | 2 | 2 | 2 | 21 | Ile β | 1 | 2 | 21 | 2 |
| Gln δ | 3 | 6 | 17 | 2 | Ile γ1 | 2 | 4 | 5 | 13 |
|  |  |  |  |  | Ile γ2 | 2 | 2 | 2 | 27 |
| Glu CO | 3 | 0 | 1 | 1 | Ile δ1 | 4 | 3 | 7 | 9 |
| Glu α | 1 | 2 | 5 | 17 |  |  |  |  |  |
| Glu β | 1 | 2 | 8 | 12 |  |  |  |  |  |
| Glu γ | 1 | 2 | 1 | 19 |  |  |  |  |  |
| Glu δ | 3 | 6 | 17 | 2 |  |  |  |  |  |
|  |  |  |  |  |  |  |  |  |  |
| Lys CO | 8 | 1 | 0 | 0 |  |  |  |  |  |
| Lys α | 1 | 2 | 14 | 6 |  |  |  |  |  |
| Lys β | 1 | 2 | 3 | 21 |  |  |  |  |  |
| Lys γ | 4 | 4 | 9 | 6 |  |  |  |  |  |
| Lys δ | 1 | 2 | 3 | 22 |  |  |  |  |  |
| Lys ε | 1 | 2 | 15 | 7 |  |  |  |  |  |
|  |  |  |  |  |  |  |  |  |  |
| Arg CO | 4 | 0 | 1 | 1 |  |  |  |  |  |
| Arg α | 1 | 2 | 4 | 11 |  |  |  |  |  |
| Arg β | 1 | 1 | 4 | 7 |  |  |  |  |  |
| Arg γ | 2 | 2 | 2 | 16 |  |  |  |  |  |
| Arg δ | 2 | 2 | 13 | 2 |  |  |  |  |  |
| Arg ζ | 11 | 17 | 7 | 3 |  |  |  |  |  |

Values are in %. Errors are estimated to 1 % for ^1^H bound ^13^C, 3 % for others. 1 % for non labeled positions is expected because of natural abundance of ^13^C. Negative values can arise because of noise in the spectra.


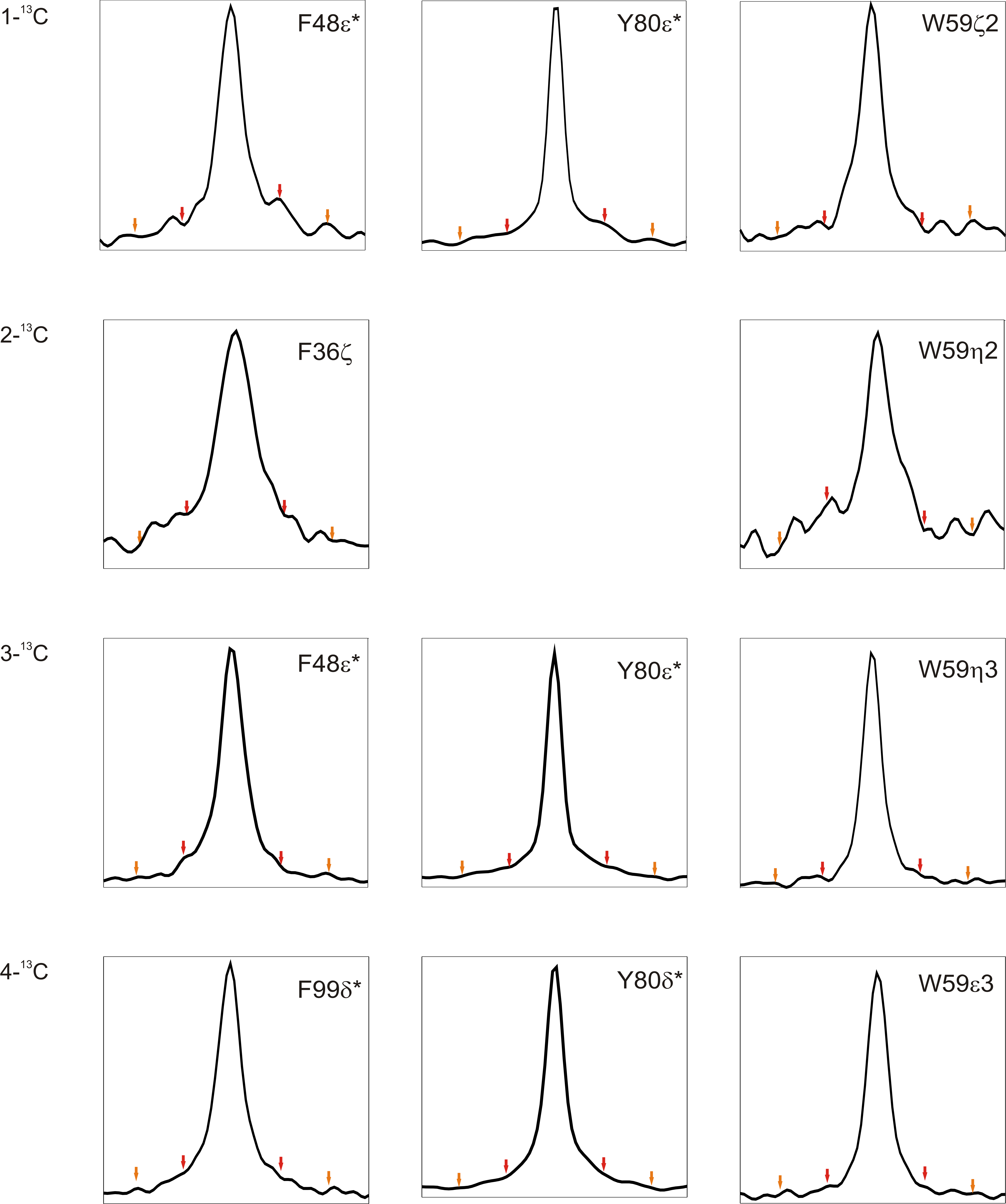


**SI Fig 1**: ^13^C slices for all aromatic positions labeled by site-selective ^13^C enriched erythrose (1-^13^C, 2-^13^C, 3-^13^C, and 4-^13^C). X-axis display 1 ppm of the corresponding ^13^C slice. Expected signals from one ^13^C neighbor (doublet) are indicated as red arrows, signals from two ^13^C neighbors (doublet of doublet) are indicated as orange arrows.


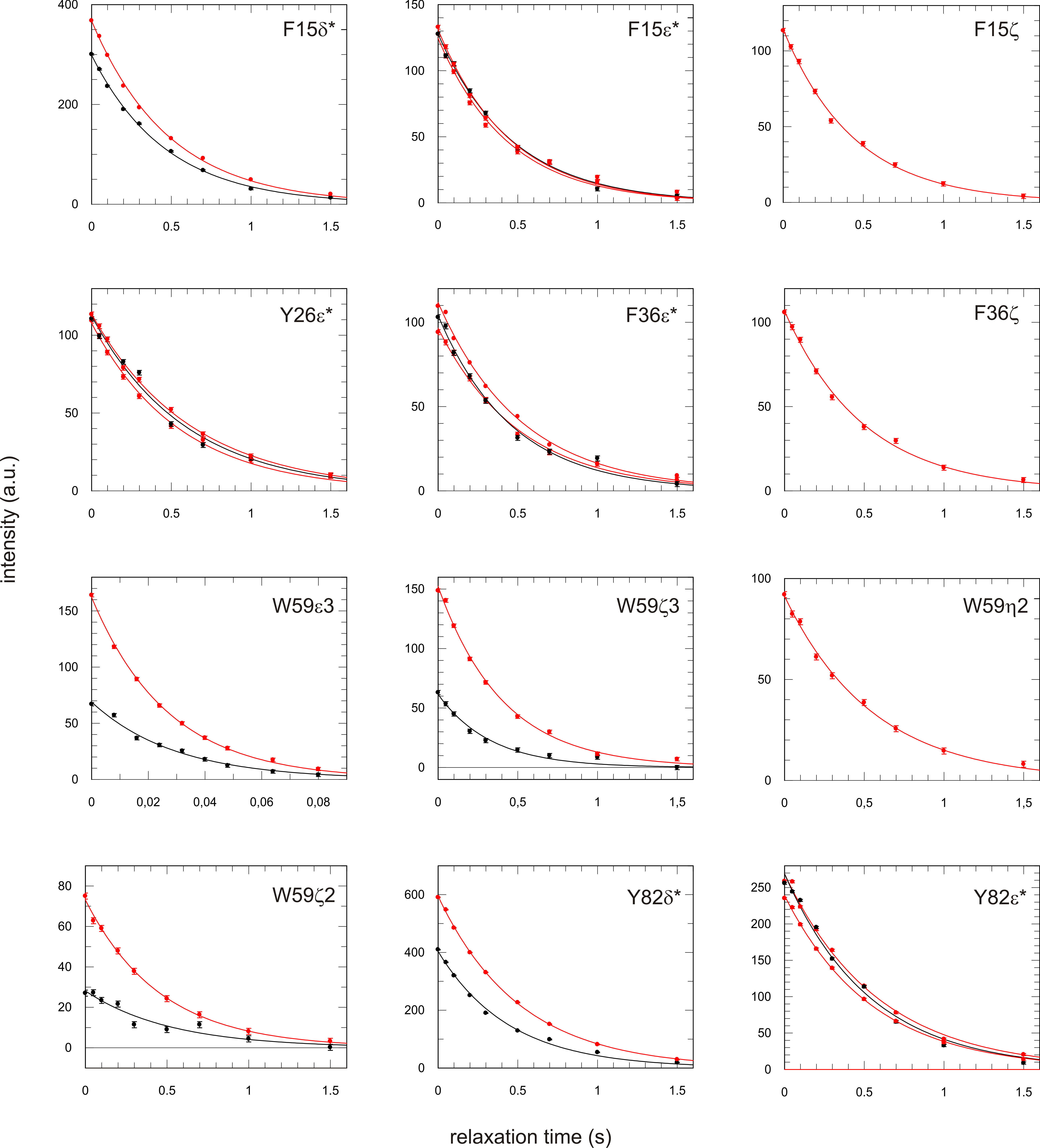


**SI Fig 2**: Aromatic ^13^C *R*_1_ curves of FKBP12 acquired at 14.1 T and 25°C. Curves from 1-^13^C or 2­-^13^C glucose labeling are shown in black, curves from 1-^13^C, 2-^13^C, 3-^13^C or 4-^13^C erythrose labeling are shown in red. Lines represent single exponential fits.


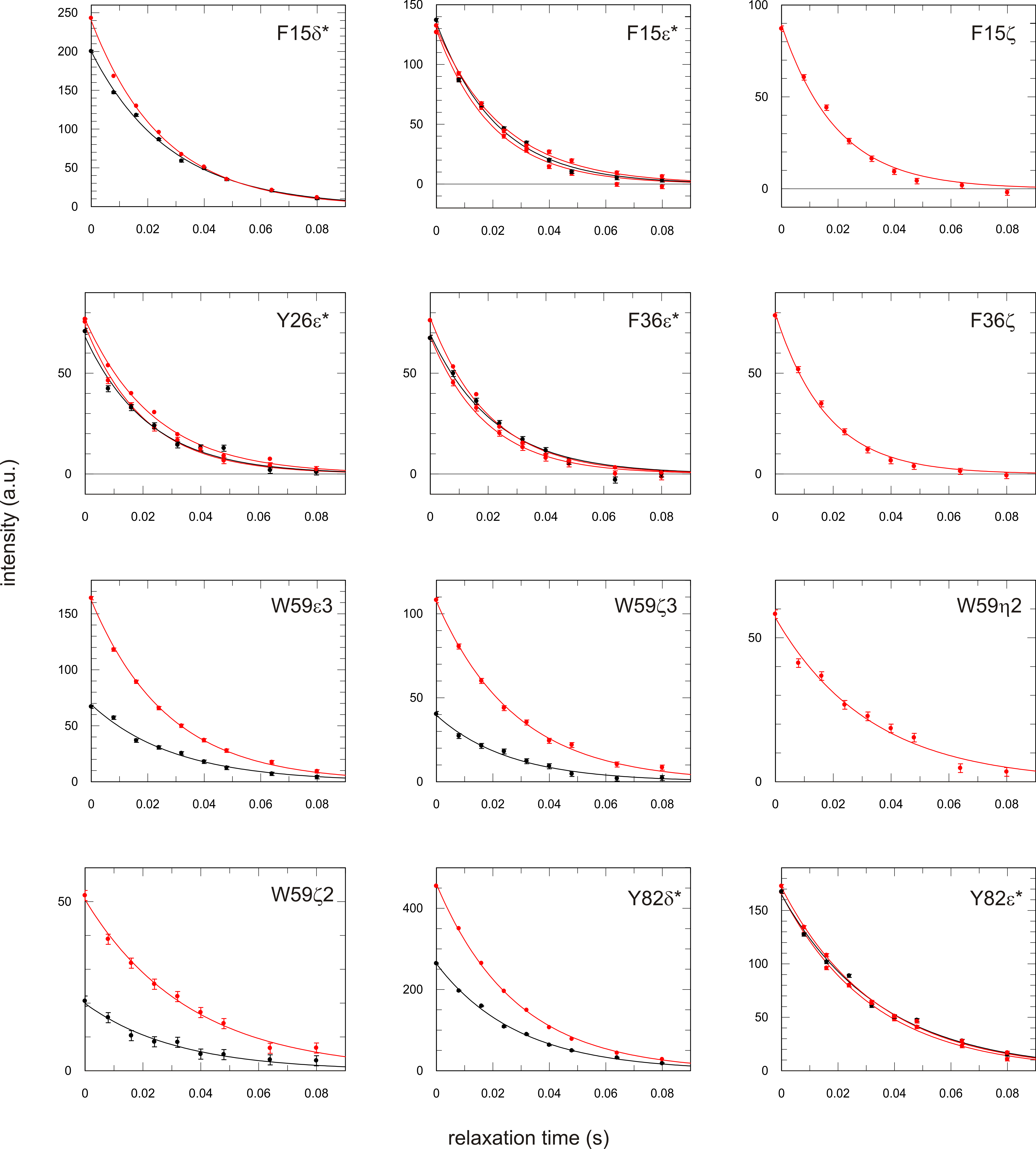


**SI Fig 3**: Aromatic ^13^C *R*_2_ curves of FKBP12 acquired at 14.1 T and 25°C. Curves from 1-^13^C or 2­-^13^C glucose labeling are shown in black, curves from 1-^13^C, 2-^13^C, 3-^13^C or 4-^13^C erythrose labeling are shown in red. Lines represent single exponential fits.
